# Cognitive Shifting Ability Does Not Predict Self-Perceived Hearing Difficulties in Adult Hearing-Aid Users

**DOI:** 10.1177/23312165261433705

**Published:** 2026-03-17

**Authors:** Francesca Molinari Luccini, Henrik Danielsson, Victoria Stenbäck, Elaine Hoi Ning Ng, Emil Holmer

**Affiliations:** 1 Disability Research Division, Department of Behavioural Sciences and Learning, 225277 Linköping University, Linköping, Sweden; 2 Division of Education, Teaching and Learning, Department of Behavioural Sciences and Learning, 225277 Linköping University, Linköping, Sweden; 3 Oticon A/S, Smørum, Denmark

**Keywords:** hearing difficulties, age-related hearing loss, shifting, hearing impairment, hearing-aid users, cognitive abilities

## Abstract

Age-related hearing loss (ARHL) often leads to hearing difficulties, impacting communication and daily functioning even among hearing-aid users. While hearing loss and cognitive functions, such as cognitive shifting ability, have been proposed as predictors of hearing difficulties, their specific contributions remain unclear. This study investigated whether hearing loss and cognitive shifting ability predict self-reported hearing difficulties across the Speech, Spatial, and Qualities of Hearing Scale questionnaire (SSQ) subscales in adults with ARHL who use hearing aids, and whether sex moderates these associations, while controlling for age and level of education. A total of 215 adults underwent audiometry, completed a cognitive flexibility task, and answered the SSQ questionnaire, of which 203 (89 females) were included in our analysis. Hierarchical multiple regression analyses revealed that less hearing loss predicted lower levels of hearing difficulties in the three subscales of the SSQ, and higher education level was a significant predictor of less reported difficulties in the Speech and Spatial subscales. Contrary to our expectations, cognitive shifting ability was not associated with hearing difficulties in any subscale, nor did sex moderate the associations between cognitive shifting ability, degree of hearing loss and hearing difficulties. The findings highlight the influence of hearing loss and education on self-reported hearing difficulties and suggest that cognitive shifting ability does not play a significant role. Future studies should explore other cognitive and demographic factors that might contribute to hearing difficulties in hearing-aid users.

## Introduction

Age-related hearing loss, or presbycusis, is a prevalent condition among older adults, characterized by a gradual decline in auditory function that typically begins at higher frequencies and progresses to lower frequencies over time (Bowl & Dawson, 2018; Huang & Tang, 2010). The impact of presbycusis includes difficulties in communication, problems with daily functioning, and psychosocial consequences, which generally increase as the condition worsens (Henderson et al., 2025). In the early stages, individuals have trouble understanding speech in noisy environments. Later, the ability to perceive and localize sounds becomes impaired, and speech comprehension might become difficult in many situations. That is, listening becomes an effortful activity (Pichora-Fuller et al., 2016), which can contribute to social isolation and depression (Bowl & Dawson, 2018; Huang & Tang, 2010). Hearing aids are a common intervention to mitigate these effects, yet it is not uncommon for hearing-aid users to continue to report difficulties in various listening environments, even while using their devices (Ferguson et al., 2017; McCormack & Fortnum, 2013). As such, hearing-aid users constitute an important group to focus on.

The diverse consequences of age-related hearing loss and experienced hearing difficulties can be conceptualized using the comprehensive International Classification of Functioning, Disability, and Health (ICF) framework (World Health Organization, 2001). Building on its biopsychosocial perspective, the ICF distinguishes between impairment of body functions and structures, activity limitations, participation restrictions, and contextual factors. In this context, hearing impairment reflects a loss of auditory function (i.e., hearing loss) to a certain extent (cf., Meyer et al., 2016), usually identified using audiometric assessments. Activity limitations describe difficulties in executing tasks, such as understanding speech in everyday listening situations, while participation restrictions refer to problems with involvement in life situations, such as engagement in social interactions and daily activities. Lastly, contextual factors refer to the environmental (e.g., listening environments) and personal factors (e.g., coping mechanisms) that might affect everyday activities and participation. Thus, while audiometry provides a measure of hearing loss, the personal burden of hearing impairment is reflected in self-reported hearing difficulties (Gatehouse & Noble, 2004; Musiek et al., 2017), a multidimensional concept encompassing functioning, activity limitations, and participation restrictions. For hearing-aid users, self-reported assessments are particularly valuable, as they capture residual hearing challenges despite amplification.

Contextual factors, in particular older age, education, and sex, influence how individuals perceive and manage hearing in different environments. For example, older age is associated with both hearing impairment and cognitive decline, which can exacerbate difficulties in complex listening situations (Choi et al., 2019; Kamil et al., 2015; Kim et al., 2017; Lin et al., 2011; Salthouse, 2009; Tsimpida et al., 2020). Higher educational attainment, on the other hand, is directly linked to improved hearing health and a greater ability to manage hearing impairment effectively (He et al., 2018; Rahimi et al., 2023). Education may also improve health outcomes by providing individuals with the knowledge and skills needed to make informed health choices (Raghupathi & Raghupathi, 2020), and act as a protective factor against cognitive decline, which is particularly relevant for older populations with age-related hearing loss (Alattar et al., 2020; Chen & Lu, 2020). Sex differences can further influence hearing outcomes via hormonal influences and structural differences in the cochlea and auditory pathways that affect auditory processing (Aloufi et al., 2023; Hämäläinen et al., 2019; Nolan, 2020). Moreover, previous studies have identified significant differences between males and females in the progression of hearing loss (Aloufi et al., 2023; Hansen et al., 2023; Nolan, 2020) and the use of hearing aids (Reavis et al., 2023). Additional factors, such as stigma related to hearing impairment and hearing aids (Liu et al., 2025), may contribute to distinct patterns of self-reported hearing difficulties between sexes. Sex differences in hearing difficulties may also be linked to cognition (Perron et al., 2022).

Cognitive abilities are known to influence how different listening environments are perceived (Marrone et al., 2019; West et al., 2022; Zekveld et al., 2013). There are also studies indicating a link between hearing loss and cognitive functions (Dryden et al., 2017; Marsja et al., 2022; Rönnberg et al., 2022). For example, based on a meta-analysis, Dryden et al. (2017) showed evidence of this in individuals with unaided hearing loss. Marsja et al. (2022), on the other hand, reported similar associations between cognitive function and speech-in-noise perception in normal-hearing listeners and hearing-aid users tested under aided conditions, by using latent-variable modeling. Furthermore, in a recent review, Shende and Mudar (2023) highlighted that hearing loss negatively impacts cognitive shifting ability, which is the ability to shift strategies during tasks (Miyake et al., 2000). In addition, Shende et al. (2021) reported that hearing loss and poorer speech-in-noise perception were associated with poorer shifting ability. This suggests a potentially challenging cycle: while hearing loss may impair shifting ability, the cognitive demands of listening in difficult environments may still partly depend on this ability. Thus, hearing-aid users who retain relatively stronger shifting ability might therefore be better able to cope with residual hearing difficulties.

To explain how hearing and cognition interact across the lifespan, several theoretical models have been proposed. For example, the common cause hypothesis posits that age-related declines in both hearing and cognition arise from shared neurobiological deterioration rather than a direct causal pathway between the two (Wayne & Johnsrude, 2015). In contrast, the information degradation hypothesis assumes that hearing loss increases the cognitive demands required for sound perception, eventually affecting broader cognitive performance. The cognitive load on perception theory emphasizes the opposite direction of influence, proposing that individuals with lower cognitive capacity are less able to compensate for hearing loss, leading to poorer sound perception outcomes. Thus, there is general agreement that declines in hearing and cognition are related in older populations, but no consensus on whether one domain influences the other, or if there is a common cause responsible for a general deterioration.

Going back to self-perceived hearing difficulties, the Framework for Understanding Effortful Listening (FUEL) is often used to describe how people allocate cognitive resources during different listening situations (Pichora-Fuller et al., 2016). According to FUEL, listening effort reflects the interplay between task demand, motivational factors, and cognitive capacity. When auditory input is degraded, such as in individuals with hearing impairment, greater cognitive effort is required for listening, and the degree of effort that the individual experiences depends on multiple cognitive resources. Thus, the FUEL explicates a link between cognitive functions and self-reported hearing difficulties.

### The Present Study

To summarize, the existing research aligns with the ICF framework by suggesting that the degree of self-reported hearing difficulties—the multidimensional construct encompassing function, activity limitation, and participation restriction—may be influenced by factors such as age, hearing loss, sex, cognitive shifting ability, and educational level. However, there is a limited understanding of how those factors interact and individually predict hearing difficulties in hearing-aid users. The level of hearing loss is presumed to increase hearing difficulties as a greater loss reduces the ability to detect and process sound, especially in complex listening environments, while shifting ability is thought to mitigate difficulties by allowing individuals to adaptively focus on relevant and ignore irrelevant aspects of the situation. In comparison to previous studies, our study makes a novel contribution by focusing exclusively on adult hearing-aid users and cognitive shifting ability. Our large sample also allows us to conduct a robust analysis of the predictors of hearing difficulties in this population. In addition, we extend previous work by investigating predictors of hearing difficulties in different listening situations, by using the three subscales of the Speech, Spatial, and Qualities of Hearing Scale questionnaire (SSQ; Gatehouse & Noble, 2004) as the outcome measures. We hypothesized that the level of hearing loss would positively predict the level of hearing difficulties and that this prediction would be stronger for females than for males, after controlling for age and level of education. Furthermore, it was hypothesized that cognitive shifting ability is associated with lower levels of hearing difficulties, especially for females (cf., Perron et al., 2022).

## Methods

### Data Collection

The variables included in this study are a selection of available variables from the N200 study described by Rönnberg et al. (2016). This is a longitudinal study examining the relationships among hearing, cognition, and speech understanding in normal hearing and hearing-impaired individuals, with data collection at the hearing clinic at Linköping University Hospital by clinical audiologists (see Rönnberg et al., 2016, for more details on data collection procedures). No new data were collected for the present analysis.

### Participants

In total, 215 individuals with hearing impairment participated in at least one session of three in the N200 study (Rönnberg et al., 2016). Eligibility criteria included bilateral, symmetrical mild-to-severe sensorineural hearing impairment, confirmed by audiometric testing, being fitted with bilateral hearing aids, and use of hearing aids for more than 1 year at the time of assessment. Participants with conductive hearing loss (difference between air conduction and bone conduction ≥10 dB HL) were excluded from the study. All participants included in the study had normal or corrected-to-normal vision (Rönnberg et al., 2016).

In the current study, 203 participants with hearing impairment using hearing aids were included in the data analysis (mean age = 61.21, *SD* = 8.05, age range: 33–80; mean years of hearing-aid use = 6.75, *SD* = 6.54), of which 89 were females. Of the original 215 participants with hearing impairment using hearing aids, we excluded those who were not an adult (i.e., younger than 18 years old) at the time they started using hearing aids (*n* = 5). Additionally, we excluded participants who had less than 1 year of hearing-aid experience (*n* = 5), those who were fitted with a unilateral hearing aid (*n* = 1), and those with missing audiometric data (*n* = 1).

### Measurement Description

The present study used the N200 data to select demographics (age, years of education, sex) and test variables on air-conduction pure tone audiometry, cognitive shifting ability, and the SSQ questionnaire (Gatehouse & Noble, 2004). Information on age, sex, and level of education was collected using a self-reported questionnaire.

#### Hearing Loss

Pure-tone audiometry for air conduction was measured at the frequencies 125, 250, 500, 750, 1,000, 2,000, 4,000, 6,000, and 8,000 Hz to assess unaided hearing thresholds. The average hearing thresholds (PTA4) were calculated at the frequencies 500, 1,000, 2,000, and 4,000 Hz, and the PTA4 of the better ear was used as a measure of the level of hearing loss in the statistical analysis. A higher PTA4 represents greater hearing loss.

#### Cognitive Shifting Ability

Cognitive shifting ability is the ability to switch between performing different tasks or mental processes during an activity or task and was tested by using a number–letter task (Rogers & Monsell, 1995). Participants were shown a series of number–letter pairs, with each pair presented in one of the four corners of a computer screen. For each pair, the participant was asked to make decisions following one of the two rules: whether the number in the pair was even or odd, or whether the letter was small or a capital letter. When pairs were shown in a corner on the upper half of the screen, decisions were made following the even/odd rule; when the pair was presented in the bottom half of the screen, decisions were instead based on the small/capital rule. At each shift between decision rules (e.g., changing from deciding whether the number is odd/even to whether the letter is small/in capital) there is a processing shifting cost reflecting the level of cognitive shifting ability (Rogers & Monsell, 1995). Before commencing the task, 16 practice trials were administered, and the participant had to have 50% correct responses on these trials for the real task to start. The first pair in the main task was presented in the upper left corner of the screen and then followed a clockwise pattern. The task included 38 pairs (18 shifting trials), and each pair stayed on the screen until the participant gave a response using one of the designated buttons, or for a maximum of 10 s. The dependent variable was defined as the shifting cost, calculated by subtracting the mean reaction time (in ms) for no-shifting trials from the mean reaction time for shifting trials. A higher score indicates poorer flexibility and slower adaptation. This variable is regarded as both a reliable and valid measure (Miyake et al., 2000), which reflects cognitive shifting ability.

#### Hearing Difficulties

The SSQ was used to assess hearing difficulties in various listening situations (Gatehouse & Noble, 2004). The SSQ is designed to evaluate functional hearing across three distinct dimensions, which are critical to understanding the impact of hearing loss on daily life. The aided-version questionnaire includes 50 questions divided into three main subscales: Speech, Spatial, and Quality.

The Speech subscale focuses on the ability to understand speech in challenging listening environments, such as in noisy settings, with competing voices, or when following fast or complex conversations (Gatehouse & Noble, 2004). The Spatial subscale evaluates the perception and localization of sounds, such as identifying the direction of a sound source, determining distance, or following the movement of sounds (Gatehouse & Noble, 2004). The Quality subscale assesses the quality, naturalness, and richness of sounds and speech, as well as the ability to distinguish different sound types, such as perceiving details in music or distinguishing voices in a group conversation (Gatehouse & Noble, 2004). Therefore, the SSQ provides a comprehensive self-reported measure of perceived hearing difficulties by focusing on activity limitations and participation restrictions associated with hearing impairment. By capturing the subjective consequences of hearing impairment on communication, social interactions, and everyday listening situations, the SSQ reflects the personal burden of hearing loss as conceptualized within the ICF framework (Meyer et al., 2016).

The participants answered all 50 questions on a scale from 0 to 10, where 10 represented “perfectly” and 0 “not at all,” reflecting on their hearing experiences while wearing hearing aids. A higher score indicates better subjective hearing performance, that is, less hearing difficulties. A recent study of the Swedish version of SSQ (Marsja et al., 2023) suggested that the reduced 12-item version of the questionnaire (SSQ12; Noble et al., 2013), but not the complete 50-question version of SSQ, fitted the hypothesized three-factor structure of the scale in confirmatory factor analysis. Therefore, the SSQ12 were used to calculate the three subscales: Speech (questions 1, 4, 10, 11, and 12); Spatial (questions 6, 9, and 13); and Quality (questions 2, 7, 9, and 14).

### Data Analysis

Statistical analyses were conducted using R version 4.5.1 (R Core Team, 2025) in RStudio version 2025.09.1 + 401 (Posit Team, 2025). First, descriptive statistics were used to describe the participants’ characteristics, predictors, and outcome measures. An independent sample *t*-test was used to assess group differences between males and females. Then, Pearson correlations between variables of interest were calculated to assess zero-order relationships. Lastly, hierarchical multiple regression analysis was conducted across nine separate models, with each model focusing on one of the three subscales of the SSQ12 questionnaire (Speech, Spatial, and Quality) as the dependent variable. For each subscale, the regression analysis was performed using blocks of predictor variables, to test our predictions: block 1: age, years of education, and sex were entered as control variables, as these demographic factors are known to influence hearing-related outcomes; block 2: cognitive shifting ability and PTA4 were added as the primary predictors of interest, enabling the evaluation of their contributions beyond demographic influences; block 3: interaction terms between sex and PTA4, and between sex and cognitive shifting ability, were included to test whether these associations varied by sex. Following inspection of the correlation analyses, an additional posthoc exploratory model was introduced to further explore observed relationships between years of education and the Speech and Spatial subscales in males: block 3b: this model included age, years of education, sex, cognitive shifting ability, PTA4, and the interaction term between sex and years of education, allowing us to assess whether the influence of education on these subscales differed between males and females. To evaluate whether each successive model provided a better fit than the previous one, changes in R-squared (Δ*R*^2^) were examined along with *F*-tests for the change in the model fit. Inferences were drawn from the model showing significant improvement over the other models while maintaining significant robustness. Assumptions of regression analysis were examined. Normality was assessed for all variables using the Kolmogorov–Smirnov test and visual inspection of *Q*–*Q* plot, and deviations were identified in the age variable and in the residuals of the Spatial and Quality subscales. To account for this, the models were additionally tested using robust regression. As the results for the predictors did not differ, we report the standard regression models for clarity and consistency.

Before performing the analyses, the dataset was examined for missing data in the Speech (*n* = 15), Spatial (*n* = 14), and Quality (*n* = 23) subscales, as well as in the shifting variable (*n* = 7) and in the education variable (*n* = 3). To address missing data in the SSQ12, shifting, and education variables, we employed multiple imputation using the Multivariate Imputation by Chained Equations approach in R, with the “mice” package (van Buuren & Groothuis-Oudshoorn, 2011). Multiple imputations (*m* = 5) were performed to account for the inherent uncertainty in the imputation process. The variables selected for imputation in the SSQ12 variable were the Speech, Spatial, and Quality subscale scores, as well as the individual scores from the 12 items. The variables used for imputation in the education variable were age and sex. Lastly, the variables used for imputation in the shifting cost variable were two other executive function variables (known to covary with shifting ability, e.g., Miyake et al., 2000) that were available in the larger dataset (i.e., updating sum and inhibition reaction time; Rönnberg et al., 2016). The missing score for PTA4 was not imputed, as it served as an exclusion criterion for participant selection.

## Results

Descriptive statistics and results for study variables are summarized in Table 1, including comparisons between males and females. Comparison between males and females revealed no statistically significant difference in age, years of education, PTA4, shifting cost, and the three subscales of the SSQ (*P* > .05). Distributions of age, years of education, PTA4, and severity of hearing impairment are illustrated in histograms provided in the Supplemental materials (Figures S1–S4).

**Table 1.** Descriptive Statistics and *t*-Test Results for Males (*n* = 114) and Females (*n* = 89) for Age, Years of Education, Unaided PTA4, Shifting Cost, and Speech, Spatial, and Quality Scores.

Variable	Females				Males				*t*	*df*	*P*	Cohen's *d*
	*M*	*SD*	*n*	Range	*M*	*SD*	*n*	Range				
Age (years)	60.6	8.08	89	37–74	61.7	8.04	114	33–80	−0.94	189	.35	−0.13
Education (years)^ª^	13.2	3.38	89	7–22.5	13.4	3.46	111	6–25.5	−0.46	191	.64	−0.07
PTA4 (dB HL)^b^	38.5	9.28	89	16.2–58.8	36.9	11.5	114	10–75	1.06	201	.29	0.15
Shifting cost (ms)^ª^	254	289	85	−434 to 1,170	329	296	111	−739 to 1,398	−1.79	183	.08	−0.26
Speech^ª^	24.5	9.58	82	5–44	25.4	10.2	106	6.2–46.5	−0.67	179	.51	−0.10
Spatialª^,b^	18.7	6.97	82	1.5–30	20.3	5.77	107	2–30	−1.68	156	.10	−0.25
Quality^ª^	27.3	7.56	74	9–40	27.2	6.86	106	9–39	0.09	147	.93	0.01

a Calculated before applying multiple imputation to account for missing data.

b Welch test is reported because Levene's test indicated that the homogeneity of variances assumption was not met for this variable.

### Correlation Analysis

Correlation analyses were conducted to explore the relationships among all study variables. The results of the correlation analysis are presented in Table 2 for all participants. Correlation analyses indicated that the SSQ12 subscales were strongly interrelated, with Pearson's *r* values ranging from .60 to .67 (*P* < .001). This pattern suggests that the subscales measure related but distinct aspects of hearing difficulties.

**Table 2.** Correlations for Continuous Study Variables for All Participants.

Variable	1	2	3	4	5	6	7
1. Speech^ª^	1						
2. Spatial^ª^	.60***	1					
3. Quality^ª^	.65***	.67***	1				
4. PTA4	−.32***	−.36***	−.29***	1			
5. Shifting cost^ª^	−.09	−.04	−.06	−.08	1		
6. Age	−.13	.02	.02	.23**	.05	1	
7. Years of education^ª^	.25**	.21**	.14	−.12	−.02	−.16*	1

a Calculated before applying multiple imputation to account for missing data.

*P < .05, **P < .01, ***P < .001.

Higher PTA4 values, reflecting greater hearing loss, were moderately associated with lower scores on the three subscales of the SSQ12, reflecting higher degrees of hearing difficulties (Speech: *r* = −.32, *P* < .001; Spatial: *r* = −.36, *P* < .001; Quality: *r* = −.29, *P* < .001). PTA4 was also positively correlated with age (*r* = .23, *P* < .01), indicating greater hearing loss with increasing age. Shifting cost was not significantly correlated with any of the subscales from SSQ12, PTA4, age, or years of education. Years of education showed small-to-moderate positive correlations with the Speech (*r* = .25, *P* < .01) and Spatial subscales (*r* = .21, *P* < .01), whereas the association with the Quality subscale did not reach statistical significance. Education was weakly negatively correlated with age (*r* = −.16, *P* < .05) and positively associated with the Speech (*r* = .25, *P* < .01) and Spatial (*r* = .21, *P* < .01) subscales.

Sex-stratified correlation analyses are presented in Table 3, with correlations for females shown in the lower left triangle and for males in the upper right triangle. Overall, the pattern of correlations showed both similarities and some differences between sexes. In both females and males, the three SSQ12 subscales (Speech, Spatial, and Quality) were strongly and positively correlated (females: *r* range = .62–.65, *P* < .001; males: *r* range = .59–.74, *P* < .001), indicating consistent associations among these domains across sexes. PTA4 was generally negatively associated with SSQ12 subscales in both groups (females: Speech: *r* = −.41, *P* < .001; Spatial: *r* = −.47, *P* < .001; Quality: *r* = −.47, *P* < .001; males: Speech: *r* = −.28, *P* < .01; Spatial: *r* = −.28, *P* < .01), but the correlation between PTA4 and the Quality subscale did not reach statistical significance in males. Additionally, PTA4 was positively correlated with age (*r* = .34, *P* < .01) and negatively associated with years of education (*r* = −.26, *P* < .05) in females, whereas this pattern was not observed in males. Shifting cost showed a small but significant negative association with the Speech subscale (*r* = −.28, *P* < .05) and years of education (*r* = −.26, *P* < .05) in females, whereas no significant associations were observed in males. Lastly, years of education were positively associated with the Speech (*r* = .31, *P* < .01) and Spatial (*r* = .25, *P* < .05) subscales in males only.

**Table 3.** Correlations for Continuous Study Variables for Female (Lower Left Triangle) and Male (Upper Right Triangle, Light Gray Shading) Participants.

Variable	1	2	3	4	5	6	7
1. Speech^ª^	1	.59***	.66***	−.28**	.01	−.16	.31**
2. Spatial^ª^	.64***	1	.74***	−.28**	.08	.09	.25*
3. Quality^ª^	.65***	.62***	1	−.19	.05	.08	.16
4. PTA4	−.41***	−.47***	−.47***	1	−.20	.18	−.05
5. Shifting cost^ª^	−.28*	−.23	−.22	.16	1	−.01	.11
6. Age	−.09	−.08	−.06	.34**	.14	1	−.14
7. Years of education^ª^	.14	.17	.13	−.26*	−.26*	−.20	1

a Calculated before applying multiple imputation to account for missing data.

*P < .05, **P < .01, ***P < .001.

### Hierarchical Multiple Regression Analysis

#### Speech Subscale

Model 2 was used to make inferences because it demonstrated a significant improvement over Model 1, while additional new predictors in Models 3 and 3b did not result in a statistically significant improvement in explained variance (see Table 4). Scatterplots of the observed data with fitted regression lines for Model 2 are provided in the Supplemental materials (Figure S5) to facilitate visual interpretation of the associations.

**Table 4.** Regression Table Speech Subscale.

Effect	β	*SE*	*t*	*P*	95% CI		*R* ^2^	Δ*R*^2^	Adj. *R*^2^	*F*	*P*
					LL	UL					
Model 1							0.05		.04	3.82	.**02**
Intercept	−.05	0.11	−0.49	.63	−0.26	0.16					
Age	−.10	0.07	−1.40	.16	−0.24	0.04					
Education	.18	0.08	2.32	.**02**	0.03	0.34					
Sex	.09	0.14	0.64	.52	−0.19	0.37					
Model 2							0.10	0.05	0.08	5.53	.**009**
Intercept	−.05	0.10	−0.44	.66	−0.25	0.16					
Age	−.05	0.07	−0.68	.50	−0.19	−0.09					
Education	.16	0.08	2.08	.**04**	0.01	0.31					
Sex	.08	0.14	0.57	.57	−0.20	0.35					
PTA4	−.21	0.08	−2.84	.**005**	−0.37	−0.07					
Shifting cost	−.10	0.07	−1.48	.14	−0.24	−0.03					
Model 3							0.11	0.01	0.08	0.27	.78
Intercept	−.06	0.11	−0.52	.60	−0.26	0.15					
Age	−.05	0.07	−0.66	.51	−0.19	0.09					
Education	.16	0.08	1.96	.06	0.00	0.32					
Sex	.09	0.14	0.61	.55	−0.19	0.36					
PTA4	−.18	0.13	−1.46	.15	−0.43	0.07					
Shifting cost	−.15	0.11	−1.37	.17	−0.37	0.07					
PTA4 × sex	−.04	0.15	−0.30	.77	−0.34	0.25					
Shifting cost × sex	.08	0.15	0.53	.60	−0.22	0.38					
Model 3b							0.12	0.02	0.09	2.39	.13
Intercept	−.05	0.10	−0.50	.61	−0.26	0.15					
Age	−.05	0.07	−0.69	.49	−0.19	0.09					
Education	.03	0.11	0.31	.76	−0.18	0.25					
Sex	.09	0.14	0.62	.54	−0.19	0.36					
PTA4	−.23	0.08	−2.97	.**004**	−0.38	−0.08					
Shifting cost	−.12	0.07	−1.73	.08	−0.26	0.02					
Education × sex	.21	0.14	1.52	.13	−0.06	0.49					

*Note*. *N* = 203. β = standardized beta; *SE* = standard error; CI = confidence interval; LL = lower limit; UL = upper limit. Results were obtained after multiple imputation and pooling of estimates. Significant *p* values are shown in bold in the table.

As predicted, PTA4 emerged as a statistically significant predictor of SSQ12 Speech subscale score in Model 2 (β = –.21, *SE* = 0.08, *t* = –2.84, *P* = .005, 95% confidence interval [CI] [–0.37, −0.07]), when controlling for age, sex, and level of education. This indicates a weak negative relationship, suggesting that a greater level of hearing loss was associated with slightly greater self-reported hearing difficulties when perceiving speech. Contrary to our expectations, cognitive shifting ability did not emerge as a predictor. In addition to PTA4, level of education emerged as a significant predictor in Model 2 (β = .16, *SE* = 0.08, *t* = 2.08, *P* = .04, 95% CI [0.01, 0.31]). Unexpectedly, the addition of interaction terms in Model 3 did not improve the model (see Table 4), suggesting that the prediction of PTA4 and cognitive shifting ability on the Speech subscale score did not differ between males and females. Similarly, Model 3b was not an improved model, and the interaction between years of education and sex was not statistically significant. This means that we did not see any statistical evidence supporting that the observed pattern in the zero-order correlations represented a moderating effect of sex on the association between level of education and the Speech subscale. In sum, PTA4 and level of education emerged as the only statistically significant predictors in the Speech subscale.

#### Spatial Subscale

Inferences for the Spatial subscale were based on Model 2, as it showed a significant improvement over Model 1, whereas the inclusion of additional predictors in Models 3 and 3b did not significantly improve the explained variance (see Table 5). Scatterplots of the observed data with fitted regression lines for Model 2 are provided in the Supplemental materials (Figure S6) to facilitate visual interpretation of the associations.

**Table 5.** Regression Table Spatial Subscale.

Effect	β	*SE*	*t*	*P*	95% CI		*R* ^2^	Δ*R*^2^	Adj. *R*^2^	*F*	*P*
					LL	UL					
Model 1							0.04		0.03	2.96	.**03**
Intercept	−.11	0.11	−1.09	.28	−0.33	0.10					
Age	.03	0.07	0.44	.66	−0.11	0.17					
Education	.18	0.07	2.45	.**02**	0.03	0.32					
Sex	.21	0.15	1.43	.15	−0.08	0.49					
Model 2							0.14	0.10	0.12	11.12	**<**.**001**
Intercept	−.10	0.10	−0.93	.35	−0.30	0.11					
Age	.10	0.07	1.44	.15	−0.04	0.24					
Education	.14	0.07	2.05	.**04**	0.00	0.27					
Sex	.17	0.14	1.21	.22	−0.11	0.45					
PTA4	−.32	0.07	−4.59	**<**.**001**	−0.46	−0.18					
Shifting cost	−.07	0.07	−0.96	.34	−0.20	0.07					
Model 3							0.15	0.01	0.12	1.63	.20
Intercept	−.09	0.10	−0.88	.38	−0.30	0.11					
Age	.11	0.07	1.55	.12	−0.03	0.25					
Education	.12	0.07	1.74	.08	−0.02	0.26					
Sex	.16	0.14	1.17	.25	−0.11	0.44					
PTA4	−.47	0.12	−3.93	**<**.**001**	−0.71	−0.23					
Shifting cost	−.11	0.11	−1.09	.27	−0.32	0.09					
PTA4 × sex	.23	0.15	1.60	.11	−0.06	0.52					
Shifting cost × sex	.11	0.14	0.81	.42	−0.17	0.39					
Model 3b							0.15	0.01	0.12	1.29	.27
Intercept	−.10	0.10	−0.98	.33	−0.30	0.10					
Age	.10	0.07	1.44	.15	−0.04	0.24					
Education	.05	0.11	0.49	.63	−0.16	0.26					
Sex	.17	0.14	1.24	.22	−0.10	0.45					
PTA4	−.33	0.07	−4.68	**<**.**001**	−0.47	−0.19					
Shifting cost	−.08	0.07	−1.14	.26	−0.22	0.06					
Education × sex	.15	0.14	1.11	.27	−0.11	0.43					

*Note*. *N* = 203. β = standardized beta; *SE* = standard error; CI = confidence interval; LL = lower limit; UL = upper limit. Results were obtained after multiple imputation and pooling of estimates. Significant *p* values are shown in bold in the table.

Similar to the results for the Speech subscale, level of education (β = .14, *SE* = 0.07, *t* = 2.05, *P* = .04, 95% CI [0.00, 0.27]) and PTA4 (β = –.32, *SE* = 0.07, *t* = –4.59, *P* < .001, 95% CI [–0.46, −0.18]) were statistically significant predictors in Model 2 also for the Spatial subscale. The coefficient for PTA4 reflects a moderate negative relationship, indicating that greater hearing loss was moderately associated with higher self-perceived hearing difficulties for localizing sounds. Cognitive shifting ability did not emerge as a significant predictor (see Table 5). This was true both for males and females, as shown by no significant improvement in Model 3 and the nonsignificant interaction terms. When adding the interaction between years of education and sex in the exploratory Model 3b, the model was not improved. Thus, for hearing difficulties in the Spatial subscale, education and hearing thresholds emerged as important predictors, corroborating the results from the Speech subscale.

#### Quality Subscale

Similarly to the Speech and the Spatial subscales, the inferences for the Quality subscale were based Model 2 (see Table 6). Scatterplots of the observed data with fitted regression lines for Model 2 are provided in the Supplemental materials (Figure S7) to facilitate visual interpretation of the associations.

**Table 6.** Regression Table Quality Subscale.

Effect	β	*SE*	*t*	*P*	95% CI		*R* ^2^	Δ*R*^2^	Adj. *R*^2^	*F*	*P*
					LL	UL					
Model 1							0.02		0.00	1.24	.36
Intercept	.01	0.12	0.08	.93	−0.24	0.26					
Age	.03	0.07	0.37	.71	−0.12	0.17					
Education	.13	0.08	1.50	.14	−0.04	0.29					
Sex	−.02	0.18	−0.10	.92	−0.39	0.35					
Model 2							0.07	0.05	0.05	6.06	.**004**
Intercept	.02	0.12	0.19	.85	−0.22	0.26					
Age	.08	0.07	1.10	.27	−0.06	0.23					
Education	.10	0.08	1.21	.23	−0.07	0.26					
Sex	−.04	0.17	−0.24	.82	−0.39	0.31					
PTA4	−.24	0.08	−3.20	.**002**	−0.39	−0.09					
Shifting cost	−.07	0.08	−0.84	.40	−0.23	0.09					
Model 3							0.09	0.02	0.06	1.49	.25
Intercept	.03	0.12	0.23	.82	−0.21	0.26					
Age	.09	0.07	1.20	.23	−0.06	0.23					
Education	.08	0.08	0.95	.35	−0.09	0.25					
Sex	−.05	0.17	−0.28	.79	−0.41	0.30					
PTA4	−.38	0.14	−2.76	.**008**	−0.66	−0.11					
Shifting cost	−.11	0.13	−0.87	.39	−0.38	0.15					
PTA4 × sex	.22	0.16	1.39	.17	−0.10	0.54					
Shifting cost × sex	.11	0.15	0.71	.48	−0.20	0.42					
Model 3b							0.08	0.01	0.05	0.66	.50
Intercept	.02	0.12	0.17	.87	−0.22	0.26					
Age	.08	0.07	1.10	.27	−0.06	0.22					
Education	.04	0.13	0.29	.78	−0.24	0.31					
Sex	−.04	0.17	−0.22	.83	−0.39	0.31					
PTA4	−.25	0.08	−3.28	.**001**	−0.40	−0.10					
Shifting cost	−.08	0.08	−0.91	.37	−0.25	0.10					
Education × sex	.10	0.16	0.65	.52	−0.21	0.41					

*Note*. *N* = 203. β = standardized beta; *SE* = standard error; CI = confidence interval; LL = lower limit; UL = upper limit. Results were obtained after multiple imputation and pooling of estimates. Significant *p* values are shown in bold in the table.

The results showed that PTA4 (β = –.24, *SE* = 0.08, *t* = –3.20, *P* = .002, 95% CI [–0.39, −0.09])—reflecting a weak positive relationship—predicted the Quality subscale scores after controlling for age, sex and level of education, whereas cognitive shifting ability (β = –.07, *SE* = 0.08, *t* = –0.84, *P* = .40, 95% CI [–0.23, 0.09]) was not a significant predictor (see Table 6). This was true for both males and females, as shown by nonsignificant interaction terms and a nonsignificant improvement in Model 3. Similarly, when the interaction between years of education and sex was added to the analysis in the exploratory Model 3b, this did not improve model fit. These findings suggest that better PTA4, rather than cognitive shifting ability, predict self-reported hearing difficulties reflected in perceiving poor sound quality.

## Discussion

The present study aimed to identify predictors of different aspects of self-perceived hearing difficulties in individuals with age-related hearing loss using hearing aids. The contributing roles of hearing loss and cognitive shifting ability, as well as the interaction between hearing loss and sex, and between cognitive shifting ability and sex, were investigated while controlling for age and years of education. Our findings revealed that hearing loss and years of education were significant predictors of hearing difficulties, although the prediction of these variables varied across different domains of hearing difficulties, as measured by the Speech, Spatial, and Quality subscales of the SSQ12 questionnaire. Individuals with less hearing loss (lower PTA4) reported lower hearing difficulties in all three subscales (Speech, Spatial, and Quality), and individuals with lower levels of education reported higher levels of hearing difficulties in the Speech and Spatial subscales. Thus, both personal and contextual factors might contribute to hearing difficulties in adult hearing-aid users.

### Hearing Difficulties are Related to Hearing Loss and Education

The correlational analysis revealed significant associations between hearing loss and self-reported hearing difficulties across all subscales of the SSQ12 questionnaire, indicating that worse hearing loss was linked to greater subjective difficulties, consistent with prior findings (Kim et al., 2017; Yang et al., 2021). Furthermore, the regression analyses suggested that lower hearing loss was associated with a lower level of hearing difficulties in all three subscales (Speech, Spatial, and Quality), after taking the effects of age, sex, education, and cognitive shifting ability into account. Previous studies have indicated that hearing loss does not always accurately reflect an individual's communication challenges, due in part to other sociodemographic and health factors influencing perceived difficulties (Choi et al., 2019; Hannula et al., 2011; Tsimpida et al., 2020). This might explain why the predictors in our regression models only covered a small proportion of variance, indicating that level of hearing loss was a weak predictor of hearing difficulties. Our findings thus support the notion that hearing loss and hearing difficulties are related but different phenomena, as defined within the ICF framework (Meyer et al., 2016; World Health Organization, 2001), and point to the existence of additional factors that influence hearing difficulties.

Educational level emerged as a significant predictor in the Speech and Spatial subscales, with individuals with higher education levels reporting a lower degree of hearing difficulties. This finding is consistent with previous research showing that higher educational attainment often leads to better health outcomes by providing individuals with the knowledge and skills needed for informed decision-making regarding their health (Raghupathi & Raghupathi, 2020). Regarding hearing loss, higher educational attainment is directly linked to improved hearing health and a greater ability to comprehend and manage the consequences of their reduced hearing effectively, thus enabling individuals to pursue preventive care (He et al., 2018; Rahimi et al., 2023). Conversely, lower educational attainment might result in limited awareness, leading to the adoption of harmful behaviors, such as smoking, poor dietary choices, and insufficient physical activity, all of which negatively impact hearing health (Chang et al., 2016; Tsimpida et al., 2019). However, we did not see an association with level of education in the Quality subscale, and one possible explanation for this is that this subscale focuses on perceptual qualities such as sound segregation, recognition, and clarity/naturalness, which may have a weaker link to education level. Nonetheless, the reason education is associated with only two of the three subscales are not fully clear, and this is a pattern that should be followed up on in future research.

Our initial study design included a three-step hierarchical regression model based on previous research and theoretical considerations. However, in the correlation analysis, we observed a potential difference in the relationships between level of education and hearing difficulties between males and females. To address this, we included an exploratory analysis, Model 3b. The interaction term between level of education and sex in this exploratory model did not improve the model and did not emerge as a significant predictor in any subscale. Thus, we saw no statistical evidence that the relationship between educational level and hearing difficulties varied between males and females, suggesting that the impact of education level on hearing difficulties is consistent across both sexes.

### No Evidence that Cognitive Shifting Ability Predicts Level of Hearing Difficulties

In the zero-order correlations, cognitive shifting ability was weakly correlated (*r* = .28) with the Speech subscale in females, and no significant correlations (*r* range = .01–.08) were found in males. Despite the zero-order association we observed in females in the present study, and contrary to our hypothesis, cognitive shifting ability was not a significant predictor of hearing difficulties in our regression models. Moreover, we found no significant association between hearing loss and cognitive shifting ability, which contrasts with our expectations based on previous studies (e.g., Shende et al., 2021) and theory (Pichora-Fuller et al., 2016). According to the FUEL (Pichora-Fuller et al., 2016), the impact of a given cognitive resource on listening outcomes depends not only on sensory and task demands but also on the individual's motivation, arousal, and allocation of cognitive capacity. It is possible that cognitive shifting ability, while relevant in some listening contexts, was not sufficiently taxed in the situations captured by our self-report measure, or that other factors, such as motivation, fatigue, or listening goals, modulated its role. Nevertheless, our findings suggest that shifting ability may not be a critical resource for managing effortful listening situations. However, this conclusion is speculative. We only included one shifting ability task, and the results might thus be task-specific. In addition, the ocular-motor component of the shifting task might have introduced an influence of undetected vestibular impairments on performance (e.g., Talkowski et al., 2005).

Importantly, shifting ability is only one of the executive functions that might impact perceived hearing difficulties. For example, Perron et al. (2022) reported that inhibitory control, which is another of the interrelated parts of the executive functions used for purposeful behaviors (Miyake et al., 2000), was correlated with the Spatial subscale in females, and with the Speech subscale in males. However, no study of the role of executive functions in self-perceived hearing difficulties, including the current one, has been properly designed to compare the relative importance of different executive functions. Future research should therefore use multiple tasks and latent-variable approaches to reduce task-specific effects in the study of how executive functions are related to the perceived difficulties in listening of adults using hearing aids.

From another perspective, the large unexplained variance in our models leaves an opening for the possibility that cognitive variables other than executive functions might be significant predictors of hearing difficulties. Notably, working memory has been consistently identified as a predictor of speech-in-noise perception, both in normal-hearing individuals and those with hearing impairment (Akashi & Martinelli, 2024; Anderson et al., 2013; Benzaquén et al., 2025). In the original sample of the N200 study, similar associations have been reported in previous studies (Rönnberg et al., 2016). One key finding from the Rönnberg et al. study was that hearing-aid users’ speech-in-noise performance could be explained by three core factors. Specifically, a “General Cognitive” factor, which heavily loaded on working memory capacity, was identified as a predictor of speech-in-noise outcomes (Rönnberg et al., 2016). Recent studies further support the relevance of working memory for self-reported hearing difficulties, though the relationships are not fully understood. For instance, a study focusing on older adults reported that auditory working memory, rather than degree of hearing loss, accounted for a substantial portion of the variability in difficulties experienced in noise and complex listening environments (Roup et al., 2025). Interestingly, another study with adults with mild to moderate hearing impairment reported that larger working memory capacity was associated with more self-reported hearing difficulties (Zekveld et al., 2013). Thus, the precise role of working memory in hearing difficulties should be investigated in future studies.

Beyond working memory, there is emerging evidence that broader cognitive functioning may be relevant for understanding perceived communication difficulties and speech-related listening challenges. For example, screening measures of general cognitive status have been linked to self-reported hearing difficulties in population-based studies (Marrone et al., 2019; West et al., 2022). Others have suggested that attention could contribute to hearing difficulties, although this has not been consistently supported (e.g., Zekveld et al., 2013). At the same time, other cognitive abilities implicated in speech-in-noise performance—such as fluid intelligence (Marsja et al., 2025; Micula et al., 2025)—may represent promising candidates for later investigation on hearing difficulties. Future research should consider multiple cognitive functions and their potentially differential contributions to real-world hearing challenges.

### No Prediction of Age and Sex on Hearing Difficulties

Age was not found to be a significant predictor of hearing difficulties. The broad age range of the participants in the present study is likely to capture diverse life experiences and biological factors contributing to hearing difficulties across different life stages. Additionally, the complexity of age-related hearing loss, which can be influenced by a combination of genetic, environmental, and lifestyle factors (Yang et al., 2023), might mean that age alone is not a sufficient predictor when other variables are considered. Moreover, it is also important to consider the role of age in the reporting of hearing difficulties. Findings from previous studies (e.g., Choi et al., 2019; Kamil et al., 2015; Kim et al., 2017; Tsimpida et al., 2020) suggest that older adults tend to report less hearing difficulties. This could be partly explained by reduced social participation, which tends to occur with increasing age (Pinto & Neri, 2017). As older individuals engage less frequently in environments with high auditory demands, they might also be less exposed to challenging listening conditions and thus report lower levels of hearing difficulties. Additionally, gradual hearing loss as an aspect of aging could contribute to subtle declines that do not directly translate into hearing difficulties as measured with self-report measurements (Lin, 2024).

Sex was also not a significant predictor of hearing difficulties. This finding contrasts with prior research that has identified that women report less hearing difficulties compared to men after adjusting for age and degree of hearing loss (Hämäläinen et al., 2019; Humes, 2021). One possible explanation for our findings is that the effect of sex on hearing difficulties may have been too small to detect within our sample size. Notably, Humes (2021) employed a larger sample, which might have provided greater statistical power to detect differences or effects related to sex. Future research using larger, more diverse samples might provide a clearer understanding of these relationships and help determine whether the absence of significant findings in our study reflects a true lack of an effect.

### Limitations and Future Directions

This study has several limitations. First, our sample consisted exclusively of adults with bilateral mild-to-severe sensorineural hearing impairment who were hearing-aid users. This improves internal validity but limits the generalizability of our findings to individuals with different types or degrees of hearing impairment or non-hearing-aid users. Moreover, our sample included participants who had been using their hearing aids for more than 1 year; however, we did not systematically track their actual usage, which may have varied considerably across individuals. Differences in hearing-aid use could influence auditory experience and adaptation, potentially affecting performance on tasks assessing hearing-related outcomes. Future research should aim to systematically monitor hearing-aid usage, for example, through datalogging, to better assess its potential impact.

Additionally, sex was self-reported, and therefore, we cannot be certain how participants interpreted the question. As biological sex—referring to the biological and physiological characteristics—and gender—representing the socially constructed norms, behaviors, and roles associated with being women and men—are distinct constructs (World Health Organization, 2021), this limitation should be considered when interpreting our findings. Lastly, measures of depression and anxiety, which are known to affect both hearing difficulties and cognitive performance (Adachi & Paul, 2024; Beaujean et al., 2013), were not included in the analysis. The absence of these variables may have influenced the observed associations. Future studies should consider incorporating such psychosocial factors to provide a more comprehensive understanding of the interplay between hearing and cognition.

## Conclusions

This study found that, in adults with hearing impairment who use hearing aids, reduced hearing difficulties were associated with less hearing loss and higher educational level. This underscores the importance of both auditory and demographic factors in shaping the subjective experiences of hearing in adult hearing-aid users. Contrary to our expectations, cognitive shifting ability was not a significant predictor of hearing difficulties in this population. This suggests that while executive functions may play a role in auditory processing, as shown in previous studies, they may not contribute to individual differences in hearing difficulties. Future research should investigate whether other cognitive domains, such as working memory, contribute to hearing difficulties in adults using hearing aids, in order to achieve a better understanding of the factors influencing hearing difficulties, beyond hearing loss alone.

## Supplemental Material

sj-docx-1-tia-10.1177_23312165261433705 - Supplemental material for Cognitive Shifting Ability Does Not Predict Self-Perceived Hearing Difficulties in Adult Hearing-Aid Users Supplemental material, sj-docx-1-tia-10.1177_23312165261433705 for Cognitive Shifting Ability Does Not Predict Self-Perceived Hearing Difficulties in Adult Hearing-Aid Users by Francesca Molinari Luccini, Henrik Danielsson, Victoria Stenbäck, Elaine Hoi Ning Ng and Emil Holmer in Trends in Hearing

## References

[bibr1-23312165261433705] Adachi R. Paul B. T. (2024). Comparison of subjective self-reported hearing and objective speech-in-noise perception as predictors of social isolation and loneliness in adults 60 years and older. International Journal of Audiology, 1–9. 10.1080/14992027.2024.2442735 39718197

[bibr2-23312165261433705] Akashi D. A. Martinelli M. C. (2024). Study of speech recognition in noise and working memory in adults and elderly with normal hearing. International Archives of Otorhinolaryngology, 28(03), e473–e480. 10.1055/s-0044-1779432 PMC11226241 38974622

[bibr3-23312165261433705] Alattar A. A. Bergstrom J. Laughlin G. A. Kritz-Silverstein D. Richard E. L. Reas E. T. Harris J. P. Barrett-Connor E. McEvoy L. K. (2020). Hearing impairment and cognitive decline in older, community-dwelling adults. The Journals of Gerontology: Series A, 75(3), 567–573. 10.1093/gerona/glz035 PMC7328194 30753308

[bibr4-23312165261433705] Aloufi N. Heinrich A. Marshall K. Kluk K. (2023). Sex differences and the effect of female sex hormones on auditory function: A systematic review. Frontiers in Human Neuroscience, 17, 10.3389/fnhum.2023.1077409 PMC10161575 37151900

[bibr5-23312165261433705] Anderson S. White-Schwoch T. Parbery-Clark A. Kraus N. (2013). A dynamic auditory-cognitive system supports speech-in-noise perception in older adults. Hearing Research, 300, 18–32. 10.1016/j.heares.2013.03.006 23541911 PMC3658829

[bibr6-23312165261433705] Beaujean A. A. Parker S. Qiu X. (2013). The relationship between cognitive ability and depression: A longitudinal data analysis. Social Psychiatry and Psychiatric Epidemiology, 48(12), 1983–1992. 10.1007/s00127-013-0668-0 23474611 PMC3906855

[bibr7-23312165261433705] Benzaquén E. Çolak H. Guo X. Lad M. Rushton S. P. Griffiths T. D. (2025). Auditory-cognitive contributions to speech-in-noise perception determined with structural equation modelling of a large sample. Scientific Reports, 15(1), 34915. 10.1038/s41598-025-18800-6 41057448 PMC12504659

[bibr8-23312165261433705] Bowl M. Dawson S. (2018). Age-Related hearing loss. Cold Spring Harbor Perspectives in Medicine, 9, a033217. 10.1101/cshperspect.a033217 PMC6671929 30291149

[bibr9-23312165261433705] Chang J. Ryou N. Jun H. J. Hwang S. Y. Song J.-J. Chae S. W. (2016). Effect of cigarette smoking and passive smoking on hearing impairment: Data from a population-based study. PLoS One, 11(1), 1–12. 10.1371/journal.pone.0146608 PMC4710530 26756932

[bibr10-23312165261433705] Chen L. Lu B. (2020). Cognitive reserve regulates the association between hearing difficulties and incident cognitive impairment evidence from a longitudinal study in China. International Psychogeriatrics, 32(5), 635–643. 10.1017/S1041610219001662 31744571

[bibr11-23312165261433705] Choi J. E. Moon I. J. Baek S.-Y. Kim S. W. Cho Y.-S. (2019). Discrepancies between self-reported hearing difficulty and hearing loss diagnosed by audiometry: Prevalence and associated factors in a national survey. BMJ Open, 9(4), e022440. 10.1136/bmjopen-2018-022440 PMC6501946 31048419

[bibr12-23312165261433705] Dryden A. Allen H. A. Henshaw H. Heinrich A. (2017). The association between cognitive performance and speech-in-noise perception for adult listeners: A systematic literature review and meta-analysis. Trends in Hearing, 21, 2331216517744675. 10.1177/2331216517744675 29237334 PMC5734454

[bibr13-23312165261433705] Ferguson M. Kitterick P. Chong L. Edmondson-Jones M. Barker F. Hoare D. (2017). Hearing aids for mild to moderate hearing loss in adults. Cochrane Database of Systematic Reviews, 9(9), CD012023. 10.1002/14651858.CD012023.pub2 PMC6483809 28944461

[bibr14-23312165261433705] Gatehouse S. Noble W. (2004). The speech, spatial and qualities of hearing scale (SSQ). International Journal of Audiology, 43(2), 85–99. 10.1080/14992020400050014 15035561 PMC5593096

[bibr15-23312165261433705] Hämäläinen A. Pichora-Fuller M. K. Wittich W. Phillips N. A. Mick P. (2019). Self-report measures of hearing and vision in older adults participating in the Canadian longitudinal study of aging are explained by behavioral sensory measures, demographic, and social factors. Ear and Hearing, 42(4), 814–831. 10.1097/AUD.0000000000000992 33741763

[bibr16-23312165261433705] Hannula S. Bloigu R. Majamaa K. Sorri M. Mäki-Torkko E. (2011). Self-reported hearing problems among older adults: Prevalence and comparison to measured hearing impairment. Journal of the American Academy of Audiology, 22(8), 550–559. 10.3766/jaaa.22.8.7 22031679

[bibr17-23312165261433705] Hansen R. S. Scheel-Hincke L. L. Jeune B. Ahrenfeldt L. J. (2023). Sex differences in vision and hearing impairments across age and European regions. Wiener Klinische Wochenschrift, 136, 55–63. 10.1007/s00508-023-02223-2 37280394

[bibr18-23312165261433705] He P. Luo Y. Hu X. Gong R. Wen X. Zheng X. (2018). Association of socioeconomic status with hearing loss in Chinese working-aged adults: A population-based study. PLoS One, 13(3), 1–12. 10.1371/journal.pone.0195227 PMC5875885 29596478

[bibr19-23312165261433705] Henderson N. Hodgson S. Mulhern B. Page K. Sampson C. (2025). A qualitative systematic review of the impact of hearing on quality of life. Quality of Life Research, 34(4), 879–892. 10.1007/s11136-024-03851-5 39579270 PMC11982117

[bibr20-23312165261433705] Huang Q. Tang J. (2010). Age-related hearing loss or presbycusis. European Archives of Oto-Rhino-Laryngology, 267(8), 1179–1191. 10.1007/s00405-010-1270-7 20464410

[bibr21-23312165261433705] Humes L. E. (2021). An approach to self-assessed auditory wellness in older adults. Ear and Hearing, 42(4), 745–761. 10.1097/AUD.0000000000001001 33720061 PMC8221726

[bibr22-23312165261433705] Kamil R. J. Genther D. J. Lin F. R. (2015). Factors associated with the accuracy of subjective assessments of hearing impairment. Ear and Hearing, 36(1), 164–167. 10.1097/AUD.0000000000000075 25158982 PMC4272625

[bibr23-23312165261433705] Kim S. Y. Kim H.-J. Kim M.-S. Park B. Kim J.-H. Choi H. G. (2017). Discrepancy between self-assessed hearing status and measured audiometric evaluation. PLoS One, 12(8), e0182718. 10.1371/journal.pone.0182718 PMC5549722 28792529

[bibr24-23312165261433705] Lin F. R. (2024). Age-related hearing loss. New England Journal of Medicine, 390(16), 1505–1512. 10.1056/NEJMcp2306778 38657246

[bibr25-23312165261433705] Lin F. R. Thorpe R. Gordon-Salant S. Ferrucci L. (2011). Hearing loss prevalence and risk factors among older adults in the United States. The Journals of Gerontology: Series A, 66A(5), 582–590. 10.1093/gerona/glr002 PMC3074958 21357188

[bibr26-23312165261433705] Liu C. Mavrommatis M. M. Govindan A. Cosetti M. K. (2025). The stigma of hearing loss: A scoping review of the literature across age and gender. Otolaryngology–Head and Neck Surgery, 172(6), 1874–1881. 10.1002/ohn.1246 40202511 PMC12120038

[bibr27-23312165261433705] Marrone N. Ingram M. Bischoff K. Burgen E. Carvajal S. C. Bell M. L. (2019). Self-reported hearing difficulty and its association with general, cognitive, and psychosocial health in the state of Arizona, 2015. BMC Public Health, 19(1), 875. 10.1186/s12889-019-7175-5 31272444 PMC6609372

[bibr28-23312165261433705] Marsja E. Holmer E. Stenbäck V. Micula A. Tirado C. Danielsson H. Rönnberg J. (2025). Fluid intelligence partially mediates the effect of working memory on speech recognition in noise. Journal of Speech, Language, and Hearing Research, 68(1), 399–410. 10.1044/2024_JSLHR-24-00465 39666895

[bibr29-23312165261433705] Marsja E. Luccini F. Stenbäck V. Holmer E. Danielsson H. (2023, August 23). Exploring the Validity of the Speech, Spatial, and Qualities of Hearing Questionnaire in Swedish: A Psychometric Analysis [Poster]. International Symposium on Auditory and Audiological Research, Nyborg Strand, Denmark.

[bibr30-23312165261433705] Marsja E. Stenbäck V. Moradi S. Danielsson H. Rönnberg J. (2022). Is having hearing loss fundamentally different? Multigroup structural equation modeling of the effect of cognitive functioning on speech identification. Ear and Hearing, 43(5), 1437–1446 10.1097/AUD.0000000000001196 34983896

[bibr31-23312165261433705] McCormack A. Fortnum H. (2013). Why do people fitted with hearing aids not wear them? International Journal of Audiology, 52(5), 360–368. 10.3109/14992027.2013.769066 23473329 PMC3665209

[bibr32-23312165261433705] Meyer C. Grenness C. Scarinci N. Hickson L. (2016). What is the International Classification of Functioning, Disability and Health and why is it relevant to audiology? Seminars in Hearing, 37(03), 163–186. 10.1055/s-0036-1584412 27489397 PMC4954783

[bibr33-23312165261433705] Micula A. Holmer E. Ning R. Danielsson H. (2025). Relationships between hearing status, cognitive abilities, and reliance on visual and contextual cues. Ear and Hearing, 46(2), 433–443. 10.1097/AUD.0000000000001596 39307930 PMC11825487

[bibr34-23312165261433705] Miyake A. Friedman N. P. Emerson M. J. Witzki A. H. Howerter A. Wager T. D. (2000). The unity and diversity of executive functions and their contributions to complex “frontal lobe” tasks: A latent variable analysis. Cognitive Psychology, 41(1), 49–100. 10.1006/cogp.1999.0734 10945922

[bibr35-23312165261433705] Musiek F. E. Shinn J. Chermak G. D. Bamiou D.-E. (2017). Perspectives on the pure-tone audiogram. Journal of the American Academy of Audiology, 28(07), 655–671. 10.3766/jaaa.16061 28722648

[bibr36-23312165261433705] Noble W. Jensen N. S. Naylor G. Bhullar N. Akeroyd M. A. (2013). A short form of the speech, spatial and qualities of hearing scale suitable for clinical use: The SSQ12. International Journal of Audiology, 52(6), 409–412. 10.3109/14992027.2013.781278 23651462 PMC3864780

[bibr37-23312165261433705] Nolan L. S. (2020). Age-related hearing loss: Why we need to think about sex as a biological variable. Journal of Neuroscience Research, 98(9), 1705–1720. 10.1002/jnr.24647 32557661

[bibr38-23312165261433705] Perron M. Dimitrijevic A. Alain C. (2022). Objective and subjective hearing difficulties are associated with lower inhibitory control. Ear and Hearing, 43(6), 1904–1916. 10.1097/AUD.0000000000001227 35544449

[bibr39-23312165261433705] Pichora-Fuller M. K. Kramer S. E. Eckert M. A. Edwards B. Hornsby B. W. Y. Humes L. E. Lemke U. Lunner T. Matthen M. Mackersie C. L. Naylor G. Phillips N. A. Richter M. Rudner M. Sommers M. S. Tremblay K. L. Wingfield A. (2016). Hearing impairment and cognitive energy: The framework for understanding effortful listening (FUEL). Ear and Hearing, 37, 5S–27S. 10.1097/AUD.0000000000000312 27355771

[bibr40-23312165261433705] Pinto J. M. Neri A. L. (2017). Trajectories of social participation in old age: A systematic literature review. Revista Brasileira de Geriatria e Gerontologia, 20(02). 10.1590/1981-22562017020.160077

[bibr41-23312165261433705] Posit Team. (2025). *RStudio: Integrated development environment for R* [Computer software]. Posit Software, PBC. http://www.posit.co/

[bibr42-23312165261433705] Raghupathi V. Raghupathi W. (2020). The influence of education on health: An empirical assessment of OECD countries for the period 1995–2015. Archives of Public Health, 78(1), 20. 10.1186/s13690-020-00402-5 32280462 PMC7133023

[bibr43-23312165261433705] Rahimi Z. Saki N. Cheraghian B. Amini P. Solaymani Dodaran M. (2023). Association between individual, household, and area-level socioeconomic status indicators and sensorineural hearing loss in adults in southwest Iran: A population-based study. Frontiers in Public Health, 11, 10.3389/fpubh.2023.1140500 PMC10150087 37139397

[bibr44-23312165261433705] R Core Team. (2025). *R: A language and environment for statistical computing* [Computer software]. R Foundation for Statistical Computing. https://www.R-project.org/

[bibr45-23312165261433705] Reavis K. M. Bisgaard N. Canlon B. Dubno J. R. Frisina R. D. Hertzano R. Humes L. E. Mick P. Phillips N. A. Pichora-Fuller M. K. Shuster B. Singh G. (2023). Sex-linked biology and gender-related research is essential to advancing hearing health. Ear and Hearing, 44(1), 10–27. 10.1097/AUD.0000000000001291 36384870 PMC10234332

[bibr46-23312165261433705] Rogers R. D. Monsell S. (1995). Costs of a predictible switch between simple cognitive tasks. Journal of Experimental Psychology: General, 124(2), 207–231. 10.1037/0096-3445.124.2.207

[bibr47-23312165261433705] Rönnberg J. Lunner T. Ng E. H. N. Lidestam B. Zekveld A. A. Sörqvist P. Lyxell B. Träff U. Yumba W. Classon E. Hällgren M. Larsby B. Signoret C. Pichora-Fuller M. K. Rudner M. Danielsson H. Stenfelt S. (2016). Hearing impairment, cognition and speech understanding: Exploratory factor analyses of a comprehensive test battery for a group of hearing aid users, the N200 study. International Journal of Audiology, 55(11), 623–642. 10.1080/14992027.2016.1219775 27589015 PMC5044772

[bibr48-23312165261433705] Rönnberg J. Signoret C. Andin J. Holmer E. (2022). The cognitive hearing science perspective on perceiving, understanding, and remembering language: The ELU model. Frontiers in Psychology, 13, 10.3389/fpsyg.2022.967260 PMC9477118 36118435

[bibr49-23312165261433705] Roup C. M. Lander D. Smith S. L. (2025). The role of auditory working memory in self-perceived hearing difficulties among older adults. American Journal of Audiology, 34(3), 516–527. 10.1044/2025_AJA-25-00024 40403336

[bibr50-23312165261433705] Salthouse T. A. (2009). When does age-related cognitive decline begin? Neurobiology of Aging, 30(4), 507–514. 10.1016/j.neurobiolaging.2008.09.023 19231028 PMC2683339

[bibr51-23312165261433705] Shende S. A. Mudar R. A. (2023). Cognitive control in age-related hearing loss: A narrative review. Hearing Research, 436, 108814. 10.1016/j.heares.2023.108814 37315494

[bibr52-23312165261433705] Shende S A Nguyen L. T Lydon E. A Husain F. T Mudar R. A (2021). Cognitive flexibility and inhibition in individuals with age-related hearing loss. Geriatrics, 6(1), 22. 10.3390/geriatrics6010022 33807842 PMC8006052

[bibr53-23312165261433705] Talkowski M. E. Redfern M. S. Jennings J. R. Furman J. M. (2005). Cognitive requirements for vestibular and ocular motor processing in healthy adults and patients with unilateral vestibular lesions. Journal of Cognitive Neuroscience, 17(9), 1432–1441. 10.1162/0898929054985419 16197696

[bibr54-23312165261433705] Tsimpida D. Kontopantelis E. Ashcroft D. Panagioti M. (2019). Socioeconomic and lifestyle factors associated with hearing loss in older adults: A cross-sectional study of the English Longitudinal Study of Ageing (ELSA). BMJ Open, 9(9). 10.1136/bmjopen-2019-031030 PMC6756470 31530617

[bibr55-23312165261433705] Tsimpida D. Kontopantelis E. Ashcroft D. Panagioti M. (2020). Comparison of self-reported measures of hearing with an objective audiometric measure in adults in the English longitudinal study of ageing. JAMA Network Open, 3(8), e2015009–e2015009. 10.1001/jamanetworkopen.2020.15009 PMC7453309 32852555

[bibr56-23312165261433705] van Buuren S. Groothuis-Oudshoorn K. (2011). Mice: Multivariate imputation by chained equations in R. Journal of Statistical Software, 45(3), 1–67. 10.18637/jss.v045.i03

[bibr57-23312165261433705] Wayne R. V. Johnsrude I. S. (2015). A review of causal mechanisms underlying the link between age-related hearing loss and cognitive decline. Ageing Research Reviews, 23(PART B), 154–166. 10.1016/j.arr.2015.06.002 26123097

[bibr58-23312165261433705] West J. S. Smith S. L. Dupre M. E. (2022). Self-reported hearing loss, hearing aid use, and cognitive function among U.S. older adults. International Journal of Population Studies, 8(1), 17–26. 10.18063/ijps.v8i1.1308 37304046 PMC10256138

[bibr59-23312165261433705] World Health Organization. (2001). *International Classification of Functioning, Disability and Health (ICF)*. https://www.who.int/standards/classifications/international-classification-of-functioning-disability-and-health

[bibr60-23312165261433705] World Health Organization. (2021, May 24). *Gender and health*. https://www.who.int/news-room/questions-and-answers/item/gender-and-health

[bibr61-23312165261433705] Yang T.-H. Chu Y.-C. Chen Y.-F. Chen M.-Y. Cheng Y.-F. Wu C.-S. Huang H.-M. (2021). Diagnostic validity of self-reported hearing loss in elderly Taiwanese individuals: Diagnostic performance of a hearing self-assessment questionnaire on audiometry. International Journal of Environmental Research and Public Health, 18(24), 13215. 10.3390/ijerph182413215 34948824 PMC8707226

[bibr62-23312165261433705] Yang W. Zhao X. Chai R. Fan J. (2023). Progress on mechanisms of age-related hearing loss. Frontiers in Neuroscience, 17, 10.3389/fnins.2023.1253574 PMC10505809 37727326

[bibr63-23312165261433705] Zekveld A. A. George E. L. J. Houtgast T. Kramer S. E. (2013). Cognitive abilities relate to self-reported hearing disability. Journal of Speech, Language, and Hearing Research, 56(5), 1364–1372. 10.1044/1092-4388(2013/12-0268) 23838985

