# Supplementary material for: Cognitive Shifting Ability Does Not Predict Self-Perceived Hearing Difficulties in Adult Hearing-Aid Users
Source: Trends Hear. 2026 Mar 17;30:23312165261433705. doi: 10.1177/23312165261433705 (PMC13009968; doi:10.1177/23312165261433705)

**Supplementary Material for: Cognitive shifting ability does not predict self-perceived hearing difficulties in adult hearing aid users**

**Figure S1**

*Histogram displaying the distribution of age*


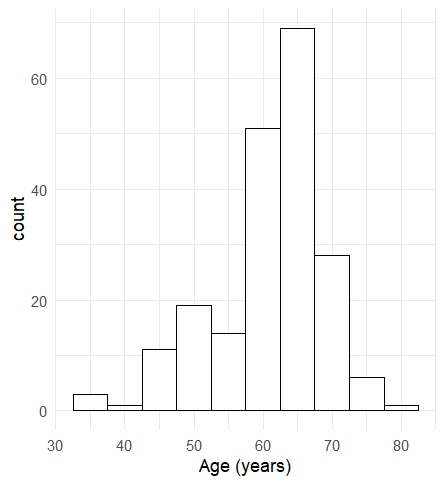


**Figure S2**

*Histogram displaying the distribution of years of education*


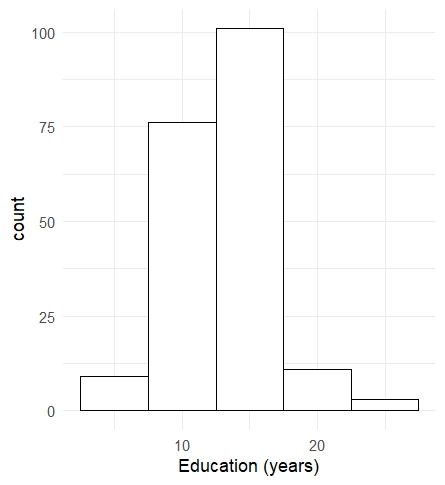


**Figure S3**

*Histogram displaying the distributions of PTA4*


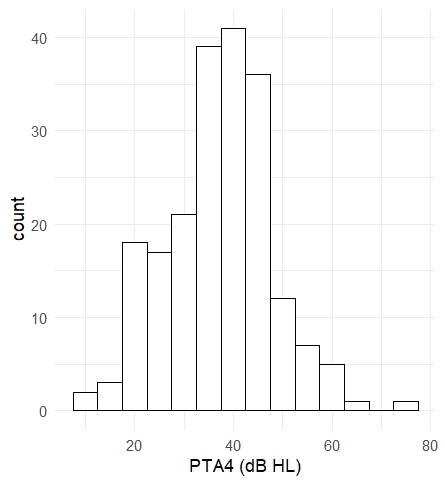


**Figure S4**

Histogram displaying the severity of hearing impairment

**
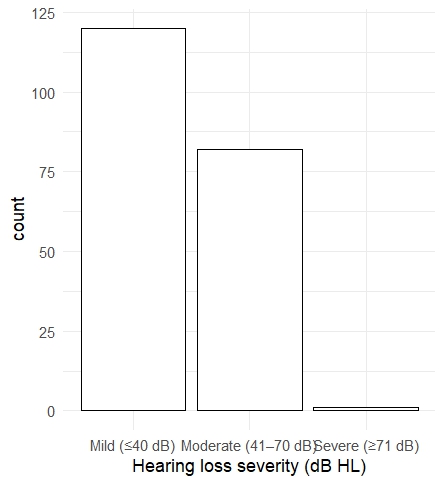
**

**Figure S5**

*Scatterplots with regression lines for Model 2 of the Speech subscale*


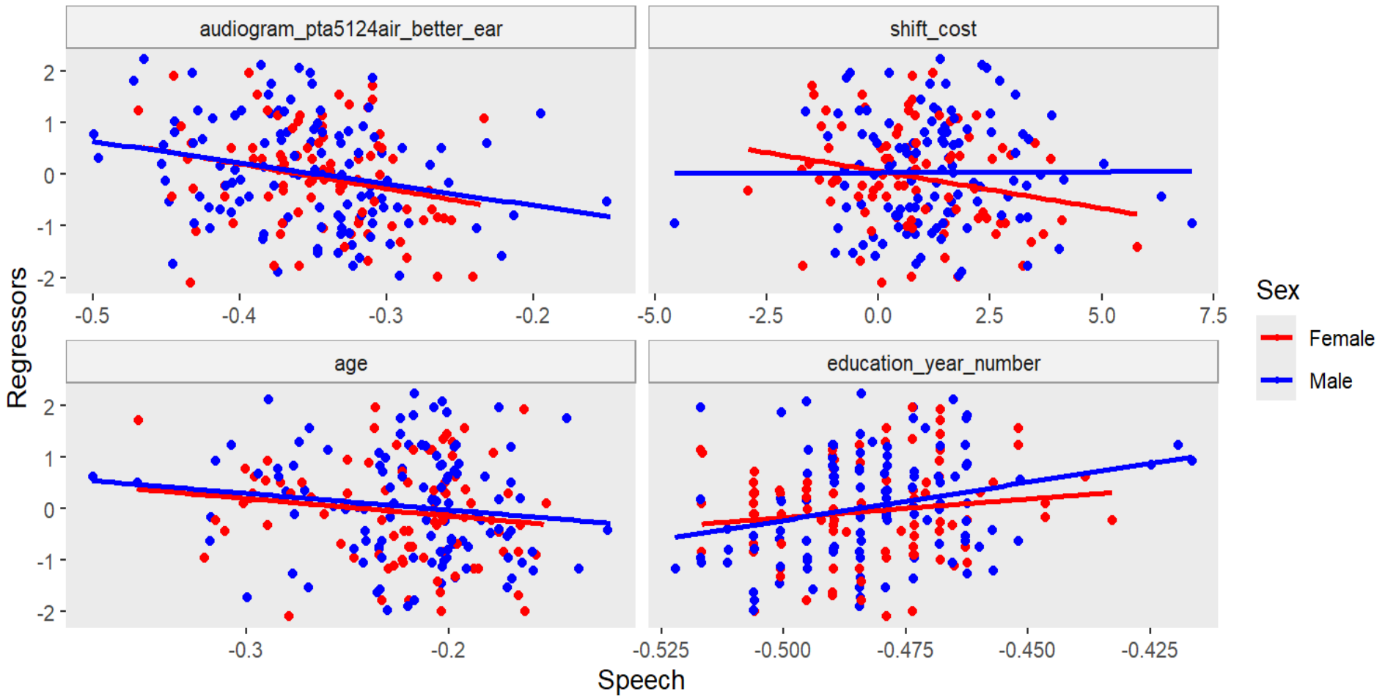


**Figure S6**

*Scatterplots with regression lines for Model 2 of the Spatial subscale*


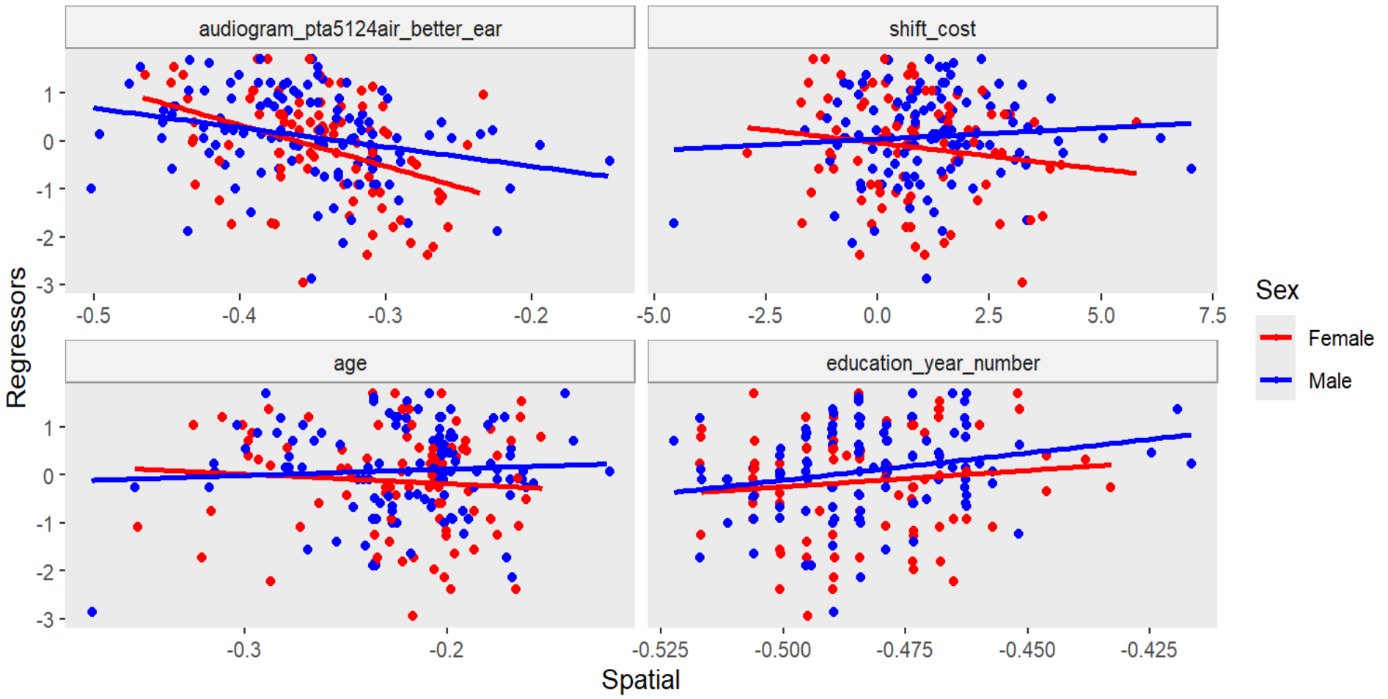


**Figure S7**

*Scatterplots with regression lines for Model 2 of the Quality subscale*


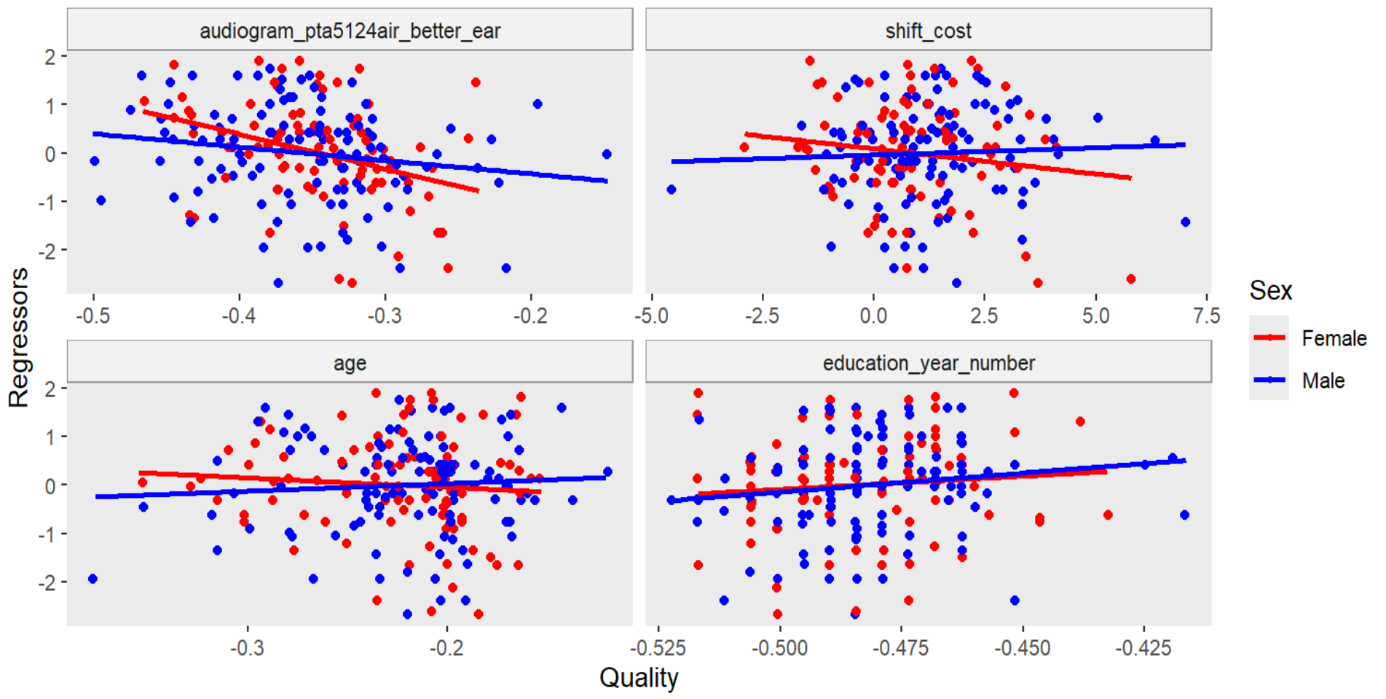

Supplement: sj-docx-1-tia-10.1177_23312165261433705 - Supplemental material for Cognitive Shifting Ability Does Not Predict Self-Perceived Hearing Difficulties in Adult Hearing-Aid Users [file sj-docx-1-tia-10.1177_23312165261433705.docx]
